# The association of plasma asprosin with anthropometric and metabolic parameters in Korean children and adolescents

**DOI:** 10.3389/fendo.2024.1452277

**Published:** 2024-10-03

**Authors:** Shin-Hee Kim, Sung Eun Kim, Yoon Hong Chun

**Affiliations:** Department of Pediatrics, Incheon St. Mary’s Hospital, College of Medicine, The Catholic University of Korea, Seoul, Republic of Korea

**Keywords:** asprosin, obesity, insulin resistance, children, mediation analysis

## Abstract

**Background:**

This study aimed to determine the correlation of plasma asprosin with anthropometric and metabolic parameters in Korean children and adolescents.

**Methods:**

This single-center study included 109 Korean children and adolescents: 62 (56.9%) obese participants with a body mass index (BMI) ≥95th percentile and 47 (43.1%) healthy controls with BMI between the 15th and 85th percentile. Metabolic parameters were measured, including fasting blood glucose, fasting insulin, homeostasis model assessment of insulin resistance (HOMA-IR), triglyceride and glucose (TyG) index, and lipid profiles.

**Results:**

Plasma asprosin levels were higher in the obese group than in the control group (mean 87.0 vs. 69.3 ng/mL; *p* = 0.001) and in the IR group than in the non-IR group (mean 98.6 vs. 70.2 ng/mL; *p* < 0.001). Plasma asprosin levels were not associated with sex or pubertal stage. Plasma asprosin levels were positively correlated with BMI SDS (*r* = 0.34; *p* = 0.002), glycated hemoglobin (HbA1c) (*r* = 0.25; *p* = 0.02), glucose (*r* = 0.33; *p* = 0.002), insulin (*r* = 0.44; *p* < 0.001), HOMA-IR (*r* = 0.47; *p* < 0.001), triglyceride (TG) (*r* = 0.33; *p* = 0.003), high-density lipoprotein (HDL) cholesterol (*r* = -0.29; *p* = 0.008), and TyG index (*r* = 0.38; *p* < 0.001). Multiple linear regression analysis indicated that plasma asprosin levels were independently associated with HOMA-IR (*p* < 0.001) and TG/HDL cholesterol ratio (*p* < 0.001).

**Conclusions:**

This study demonstrated an association between plasma asprosin levels and obesity and insulin resistance in Korean children and adolescents.

## 1 Introduction

Obesity is defined as an accumulation of excess body fat. Adipose tissue serves as an energy storage organ and secretes lipids, peptides, cytokines, and adipokines that regulate the metabolism of the liver, muscles, heart, and central nervous system. Childhood obesity has become one of the more significant public health issues worldwide (1), and it is strongly associated with type 2 diabetes, a high risk of cardiovascular diseases, and metabolic syndrome in adulthood (2).

Asprosin has been identified as a fasting-induced protein hormone that modulates hepatic glucose release, and it is cleaved from the C-terminal end of profibrillin (encoded by FBN1). Increased asprosin levels in the liver trigger cAMP and PKA signaling pathways, resulting in higher glucose release from the liver to circulation (3). Human adult studies have shown increased circulating asprosin levels in patients with obesity (4), insulin resistance (3, 5–7), metabolic syndrome (8), type 2 diabetes mellitus (9, 10), or gestational diabetes mellitus (11). Ugur and Aydın reported that serum and saliva asprosin levels positively correlated with body mass index (BMI) (12). Experimental animal studies showed that neutralizing plasma asprosin using a monoclonal antibody reduces appetite and body weight in obese mice and improves their glycemic profile (13, 14). Wang et al. demonstrated that asprosin levels decreased significantly six months after bariatric surgery in the morbidly obese patients (15). In young women with low physical activity and visceral obesity, an 8-week exercise program improved BMI and insulin resistance, decreasing asprosin levels (16).

Despite these findings, only limited studies have evaluated the relationship between asprosin and obesity in children (17–20). Evaluation of the linkage between asprosin and obese children and adolescents who are not suffering from long-lasting obesity-related complications could broaden knowledge about the mechanisms underlying asprosin pathophysiology. The current study aimed to evaluate the relationship of asprosin levels with anthropometric and metabolic parameters in Korean children and adolescents.

## 2 Materials and methods

### 2.1 Subjects

**Figure 1:**
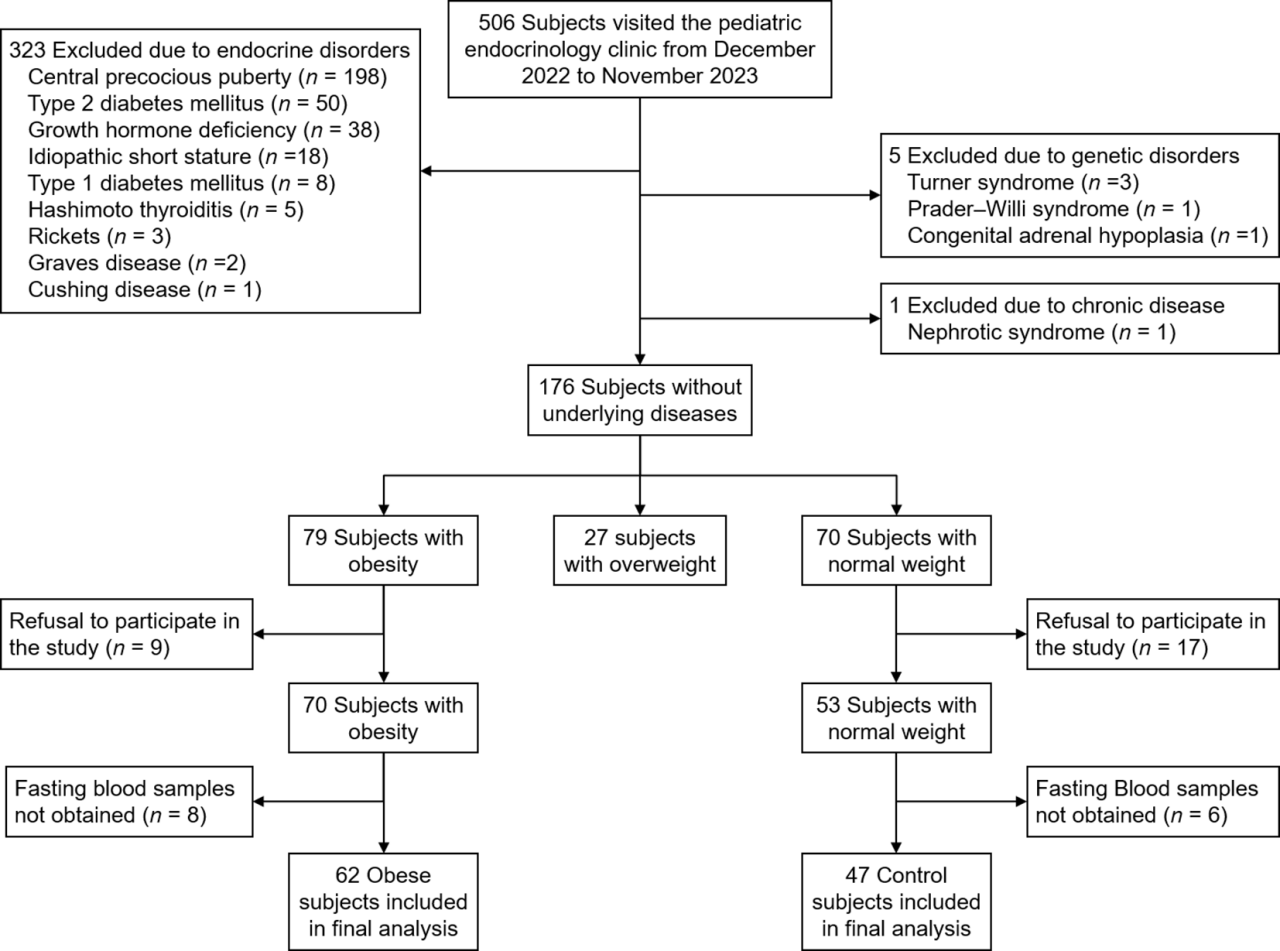
Flowchart of inclusion and exclusion criteria for this study.

All children and adolescents who visited the pediatric endocrinology clinic of Incheon St. Mary’s Hospital from December 2022 to November 2023 were screened for inclusion and exclusion criteria. Inclusion criteria were children and adolescents with obesity (defined as BMI ≥ 95th percentile for age and sex). Normal-weight subjects (defined as 15th ≤ BMI < 85th percentile for age and sex) were included as controls. Exclusion criteria were children with endocrine disorders (central precocious puberty, diabetes mellitus, growth hormone deficiency, idiopathic short stature, thyroid disease, rickets, Cushing disease), genetic disorders (Turner syndrome, Prader-Willi syndrome, congenital adrenal hypoplasia), and other chronic diseases (liver disease other than metabolic dysfunction-associated steatotic liver disease [MASLD], renal disease). Subjects whose parents refused to participate in the study or those from whom fasting blood samples were not obtained were excluded. **Figure 1** shows the flowchart of inclusion and exclusion criteria for the study. Written informed consent was obtained from all participants and their parents. The study was approved by the Institutional Review Board of Incheon St. Mary’s Hospital (IRB number: OC22OISI0140).

### 2.2 Anthropometry measurements

Height was recorded to the nearest 0.1 cm using a Harpenden Stadiometer, and weight was recorded to the nearest 0.1 kg using an electronic scale. SDS for weight, height, and BMI were calculated according to age and sex using the reference data published in the 2017 Korean National Growth Charts (21). The pubertal stage was assessed by the pediatric endocrinologist according to Marshall and Tanner standards (22).

### 2.3 Laboratory evaluations

Venous blood samples were obtained in the morning after a 10-hour overnight fast. Serum samples were analyzed for HbA1c, glucose, insulin, total cholesterol, TG, low-density lipoprotein (LDL) cholesterol, high-density lipoprotein (HDL) cholesterol, aspartate transaminase (AST), alanine transaminase (ALT), uric acid, 25(OH)-vitamin D, and asprosin. In children with elevated ALT, measurements were taken every 3 months. If ALT was consistently more than twice the upper normal limit, further evaluation was conducted to exclude other causes of liver disease. Glycated hemoglobin (HbA1c) levels were measured using automated high-performance liquid chromatography (HLC-723 G7, Tosoh, Tokyo, Japan). Serum insulin levels were measured with Elecsys insulin Assay (Cobas e411 immunoassay analyzer; Roche Diagnostics, Mannheim, Germany). Serum glucose, total cholesterol, LDL cholesterol, HDL cholesterol, TG, AST, and ALT were tested using a Beckman Coulter AU5800 clinical chemistry analyzer and the manufacturer’s reagents (Beckman Coulter, Brea, CA, USA). Serum 25-hydroxy vitamin D was measured with a Siemens ADVIA Centaur^®^ vitamin D TOTAL immunoassay (Siemens Healthcare, Erlangen, Germany).

Blood samples for the measurement of plasma asprosin were collected using EDTA tubes and centrifuged at 2,000 × g for 10 minutes at room temperature. The isolated plasma samples were stored at -80°C until asprosin levels were measured. Plasma samples were diluted 1:2 with sample diluent, and asprosin levels were measured according to the manufacturer’s protocol using commercial ELISA kits (Cat. No. ab275108, ELISA sandwich kit, Abcam, USA). The manufacturer reported that the intra-assay coefficient of variation is 7.9% and the minimum detectable concentration is 0.92 ng/mL.

### 2.4 Definitions

Dyslipidemia was defined as either or a combination of total cholesterol ≥200 mg/dL, LDL cholesterol ≥ 130 mg/dL, HDL cholesterol ≤40 mg/dL, and TG ≥150 mg/dL according to the Third Report of the National Cholesterol Education Program (23) and the American Diabetes Association (24). Insulin resistance was estimated from fasting glucose and insulin serum levels using the homeostasis model assessment of insulin resistance (HOMA-IR) as fasting insulin (µU/ml) × fasting glucose (mmol/L)/22.5. Insulin resistance was defined as a HOMA-IR >2.5 in pre-pubertal and >4.0 in pubertal subjects (25). The TyG index was calculated as ln (fasting triglycerides [mg/dL] × fasting plasma glucose [mg/dL]/2).

### 2.5 Statistical analysis

All analyses were performed using R statistical software (version 4.3.2, R Foundation for Statistical Computing, Vienna, Austria). Categorical variables are presented as numbers (%), and continuous variables are presented as mean ± standard deviation. Chi-square tests or Fisher’s exact tests were employed for categorical variables between two groups, as appropriate. The Student’s t-test and Mann-Whitney U test were utilized for continuous variables with normal and non-normal distributions, respectively. The normality of distribution was assessed using the Shapiro-Wilk test. The correlation between asprosin and continuous variables was examined using Pearson’s correlation and partial correlation analysis. Multiple linear regression was used to estimate the independent association between asprosin levels and clinical parameters. Age, sex, and variables that were statistically significant in simple linear regression were included in the multiple linear regression analysis. Mediation analysis was conducted to examine whether the association between BMI-SDS and HOMA-IR was mediated by asprosin. All *p*-values < 0.05 were considered statistically significant.

## 3 Results

### 3.1 Clinical characteristics of obese and normal weight groups

**Table 1** shows the clinical and biochemical characteristics in the obese and normal weight groups. Of 109 children and adolescents, 62 (56.9%) were obese and 47 (43.1%) were normal weight (**Figure 1**). The mean age was 9.6 years (median, 9.0; range, 6–14). Male sex was more common in the obese group than in the control group (59.7 vs. 31.9%; *p* = 0.007). Height SDS, glucose, insulin, HOMA-IR, total cholesterol, and ALT were higher in the obese group than in the control group. Insulin resistance was more common in the obese group than in the control group (58.1% vs. 2.1%; *p* < 0.001). Dyslipidemia was more common in the obese group than in the control group (59.7% vs. 36.2%; *p* = 0.03). There were no significant differences in systolic blood pressure, diastolic blood pressure, Tanner stage, HbA1c, TG, LDL cholesterol, HDL cholesterol, TG/HDL cholesterol ratio, TyG index, AST, uric acid, and 25(OH)-vitamin D levels.

**Table 1:** Demographic, clinical, and laboratory data for obese and control subjects.

Characteristic	Obese (n = 62)	Control (n = 47)	*p-*value
Age, years			
Mean	9.3 ± 2.0	9.9 ± 1.8	0.10
Median (range)	9.0 (8.0–11.0)	9.0 (9.0–11.0)	0.08
Male sex	37 (59.7)	15 (31.9)	0.007
Height SDS	1.2 ± 0.9	0.3 ± 1.0	<0.001
Weight SDS	2.0 ± 0.3	0.8 ± 0.6	<0.001
BMI, kg/m^2^	24.9 ± 2.3	20.2 ± 1.3	<0.001
BMI SDS	2.2 (2.1–2.4)	0.8 (0.7–0.9)	<0.001
Systolic BP, mmHg	100 (100–112)	100 (100–105)	0.33
Diastolic BP, mmHg	60 (60–65)	65 (60–65)	0.44
Tanner stage			0.53
1	21 (33.9)	9 (19.1)	
2	25 (40.3)	24 (51.1)	
3	9 (14.5)	8 (17.0)	
4	5 (8.1)	4 (8.5)	
5	2 (3.2)	2 (4.3)	
Puberty	41 (66.1)	38 (80.9)	0.14
HbA1c, %	5.5 (5.3–5.6)	5.3 (5.2–5.4)	0.10
Glucose, mg/dL	94.5 ± 8.2	89.5 ± 8.8	0.02
Insulin, μU/mL	17.5 (13.1–22.6)	7.3 (5.8–8.6)	<0.001
HOMA-IR	4.0 (2.9–5.1)	1.7 (1.3–1.9)	<0.001
Insulin resistance	36 (58.1)	1 (2.1)	<0.001
Total cholesterol, mg/dL	163 ± 32	148 ± 10	0.001
Triglycerides, mg/dL	120 ± 38	110 ± 42	0.32
LDL cholesterol, mg/dL	107 ± 25	110 ± 27	0.68
HDL cholesterol, mg/dL	49 (41–53)	49 (47–63)	0.21
TG/HDL cholesterol ratio	2.4 (1.7–3.2)	1.7 (1.5–2.8)	0.09
Dyslipidemia	37 (59.7)	17 (36.2)	0.03
TyG index	9.3 ± 0.4	9.1 ± 0.4	0.11
AST, U/L	22 (19–27)	20 (17–26)	0.20
ALT, U/L	22 (17–27)	15 (14–20)	0.006
Uric acid, mg/dL	5.1 ± 1.3	4.6 ± 1.0	0.10
25(OH)-vitamin D, ng/mL	13.8 (6.7–18.4)	14.5 (7.0–18.6)	0.98
Asprosin, ng/mL	87.0 ± 26.2	69.3 ± 16.2	0.001

For categorical variables, data are presented as the number of patients (%). For continuous variables, data are presented as the mean ± standard deviation for parametric variables or as the median (interquartile range) for nonparametric variables.

AST, aspartate transaminase; ALT, alanine transaminase; BMI, body mass index; HDL, high-density lipoprotein; HOMA-IR, homeostasis model assessment-insulin resistance; LDL, low-density lipoprotein; SDS, standard deviation score; TyG index, triglyceride and glucose index.

### 3.2 Correlation of plasma asprosin with patient characteristics

**Figure 2:**
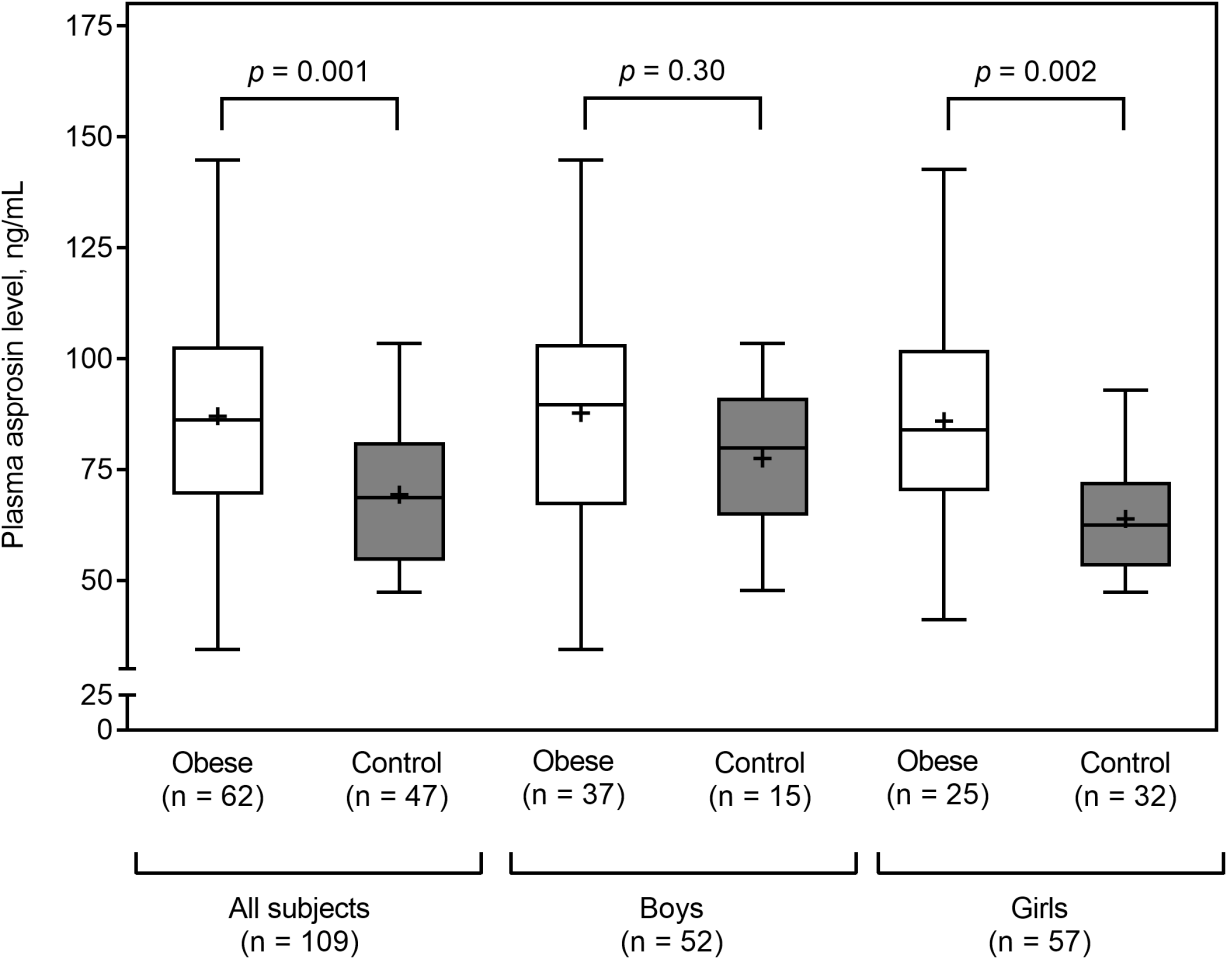
Plasma asprosin between obese and control (normal weight) groups by sex. The box and whisker plots indicate the median (horizontal line in the box), mean (black cross), 25^th^ percentile (bottom line of the box), 75^th^ percentile (top line of the box), and 5^th^ and 95^th^ percentiles (whiskers).

**Figure 3:**
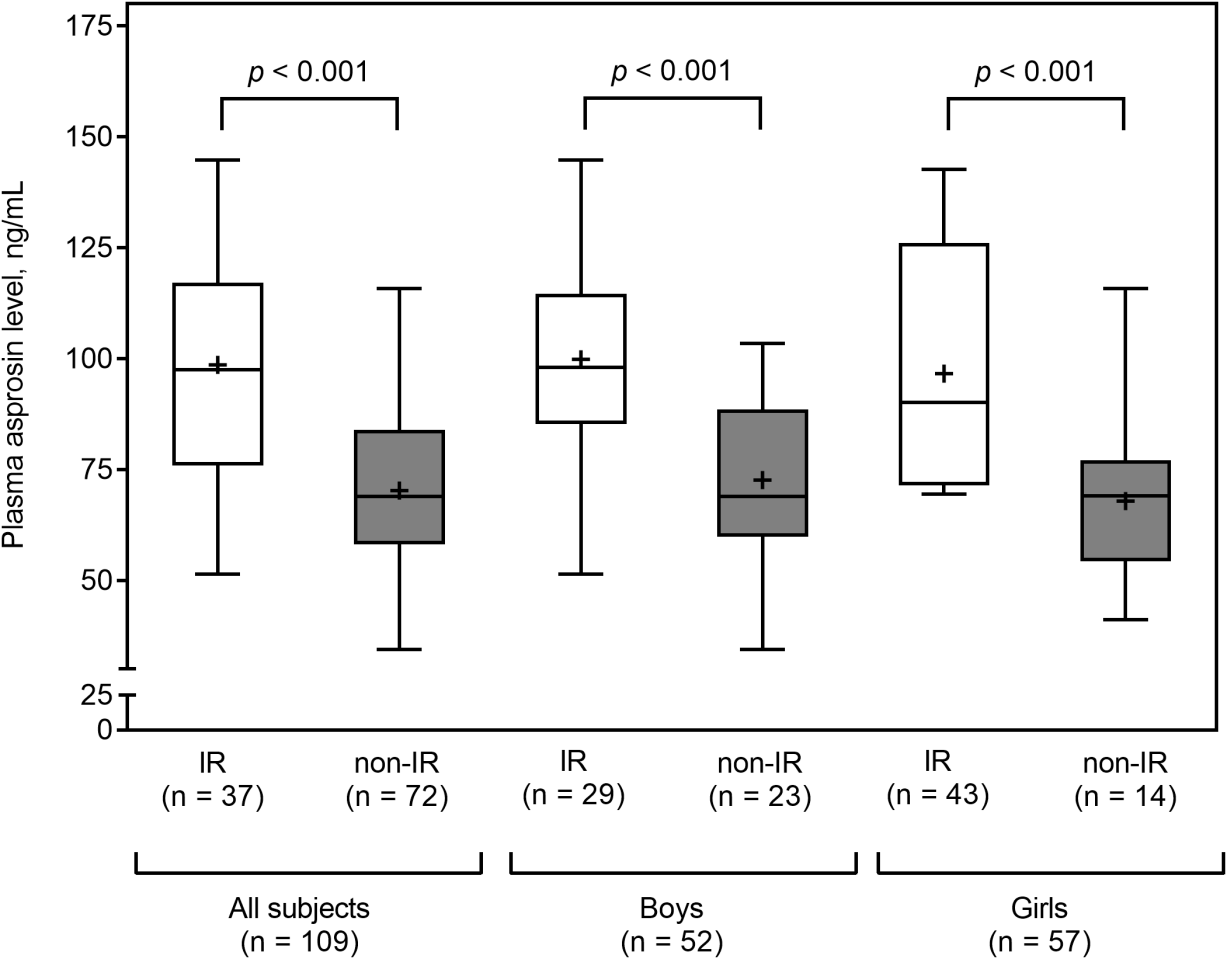
Plasma asprosin between subjects with or without insulin resistance (IR) stratified by sex. The box and whisker plots indicate the median (horizontal line in the box), mean (black cross), 25^th^ percentile (bottom line of the box), 75^th^ percentile (top line of the box), and 5^th^ and 95^th^ percentiles (whiskers).

Plasma asprosin levels did not differ between boys and girls (median 87.6 vs. 72.6; *p* = 0.14). Obese subjects had higher plasma asprosin levels than healthy subjects (mean 87.6 ± 26.2 vs. 69.3 ± 16.3 ng/mL; *p* = 0.001). This finding was not evident in boys (mean 87.8 ± 26.4 vs. 77.5 ± 17.6 ng/mL; *p* = 0.30), but it was evident in girls (mean 85.9 ± 26.4 vs. 63.9 ± 13.3 ng/mL; *p* = 0.002) (**Figure 2**). Plasma asprosin levels were higher in the insulin resistance group than in the non-insulin resistance group (mean 98.6 ± 24.6 vs. 70.2 ± 17.7 ng/mL; *p* < 0.001). This finding was evident in both boys (mean 99.8 ± 24.5 vs. 72.6 ± 17.9 ng/mL; *p* < 0.001) and girls (mean 96.7 ± 25.7 vs. 67.8 ± 17.6 ng/mL; *p* < 0.001) (**Figure 3**). There was no difference in plasma asprosin levels between subjects with and without dyslipidemia (mean 86.4 ± 29.0 vs. 78.4 ± 19.6 ng/mL; *p* = 0.14).

### 3.3 Correlations of plasma asprosin and clinical and biochemical parameters

**Table 2** shows the simple correlation analysis between plasma asprosin and clinical and biochemical parameters. Plasma asprosin levels were positively correlated with BMI SDS (*r* = 0.34; *p* = 0.002), HbA1c (*r* = 0.25; *p* = 0.02), fasting glucose (*r* = 0.33; *p* = 0.002), fasting insulin (*r* = 0.44; *p* < 0.001), HOMA-IR (*r* = 0.47; *p* < 0.001), and TyG index (*r* = 0.38; *p* < 0.001). Plasma asprosin levels were positively correlated with TG (*r* = 0.33; *p* = 0.003) and negatively correlated with HDL cholesterol (*r* = -0.29; *p* = 0.008). Plasma asprosin level was positively correlated with TG/HDL cholesterol ratio (*r* = 0.42; *p* < 0.001). The positive correlation between plasma asprosin and BMI SDS was evident in the insulin resistance group (*r* = 0.39; *p* = 0.02) but not in the non-insulin resistance group (*r* = 0.05; *p* = 0.76).

**Table 2:** Simple correlations between asprosin levels and clinical and biochemical parameters.

Characteristic	*r*	*p*-value
Age, years	-0.19	0.09
BMI SDS	0.34	0.002
Systolic BP, mmHg	0.13	0.26
Diastolic BP, mmHg	0.12	0.29
Tanner stage	-0.17	0.13
HbA1c, %	0.25	0.02
Glucose, mg/dl	0.33	0.002
Insulin, μU/mL	0.44	<0.001
HOMA-IR	0.47	<0.001
Total cholesterol, mg/dl	0.07	0.52
Triglycerides, mg/dl	0.33	0.003
LDL cholesterol, mg/dl	0.02	0.89
HDL cholesterol, mg/dl	-0.29	0.008
TG/HDL cholesterol ratio	0.42	<0.001
TyG index	0.38	<0.001
AST, U/L	0.03	0.79
ALT, U/L	0.07	0.52
Uric acid, mg/dl	0.22	0.05
25(OH)-vitamin D, ng/mL	-0.19	0.12

AST, aspartate transaminase; ALT, alanine transaminase; BMI, body mass index; BP, blood pressure; HDL, high-density lipoprotein; HOMA-IR, homeostasis model assessment-insulin resistance; LDL, low-density lipoprotein; SDS, standard deviation score; TyG index, triglyceride and glucose index.

After adjusting for age and BMI SDS, glucose (*r* = 0.27; *p* = 0.02), insulin (*r* = 0.34; *p* = 0.002), HOMA-IR (*r* = 0.37; *p* < 0.001), TG (*r* = 0.27; *p* = 0.02), HDL cholesterol (*r* = -0.26; *p* = 0.02), TG/HDL cholesterol ratio (*r* = 0.37; *p* < 0.001), and TyG index (*r* = 0.31; *p* = 0.005) were significantly correlated with asprosin (**Table 3**). When adjusting for age within the same sex, the correlation of asprosin with insulin, HOMA-IR, and the TG/HDL cholesterol ratio was evident in both boys and girls. The correlation of asprosin with glucose and TyG index was evident in boys but not in girls. In contrast, the correlation of asprosin with uric acid and 25(OH)-vitamin D was evident in girls but not in boys (**Table 3**).

**Table 3:** Partial correlations between asprosin levels and clinical and biochemical parameters^a^.

Characteristic	All subjects (n =109)		Boys (n =52)		Girls (n =57)	
	*r*	*p*-value	*r*	*p*-value	*r*	*p*-value
Systolic BP	0.09	0.45	0.13	0.42	-0.01	0.95
Diastolic BP	0.19	0.10	0.20	0.19	0.08	0.66
Tanner stage	-0.03	0.77	0.02	0.90	-0.16	0.36
HbA1c	0.21	0.06	0.21	0.17	0.30	0.08
Glucose	0.27	0.02	0.37	0.01	0.15	0.40
Insulin	0.34	0.002	0.41	0.006	0.34	0.045
HOMA-IR	0.37	<0.001	0.48	0.001	0.35	0.04
Total cholesterol	-0.03	0.81	-0.01	0.97	-0.08	0.65
Triglycerides	0.27	0.02	0.33	0.03	0.22	0.21
LDL cholesterol	0.00	0.99	0.02	0.91	-0.08	0.63
HDL cholesterol	-0.26	0.02	-0.26	0.09	-0.22	0.20
TG/HDL cholesterol ratio	0.37	<0.001	0.38	0.01	0.37	0.03
TyG index	0.31	0.005	0.38	0.01	0.24	0.17
AST	-0.05	0.64	-0.12	0.43	0.05	0.78
ALT	-0.01	0.90	-0.14	0.36	0.16	0.34
Uric acid	0.21	0.06	0.01	0.96	0.51	0.002
25(OH)-vitamin D	-0.23	0.06	-0.12	0.48	-0.46	0.009

a Partial correlations after adjustment for age and BMI SDS.

AST, aspartate transaminase; ALT, alanine transaminase; BMI, body mass index; BP, blood pressure; HDL, high-density lipoprotein; HOMA-IR, homeostasis model assessment-insulin resistance; LDL, low-density lipoprotein; SDS, standard deviation score; TyG index, triglyceride and glucose index.

**Figure 4:**
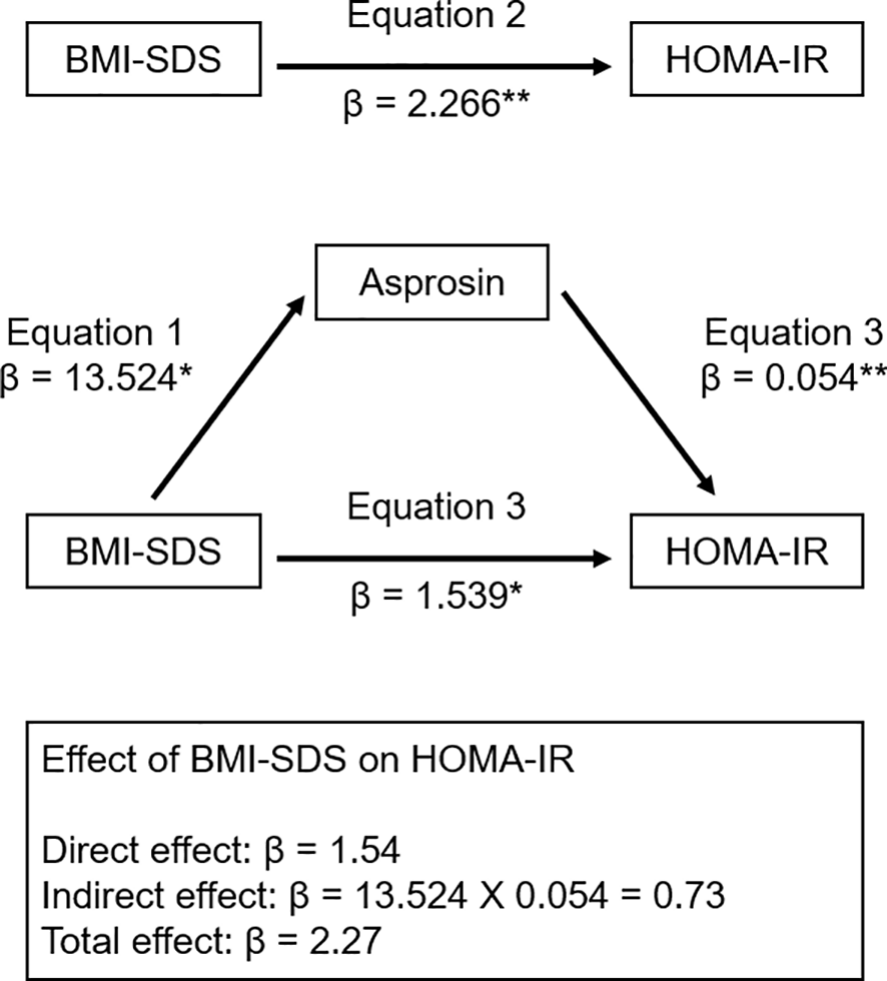
Mediation model of the relationship of BMI SDS and HOMA-IR. β indicates the standardized coefficient. *p < 0.01; ** *p* < 0.001. BMI SDS, body mass index standard deviation score; HOMA-IR, homeostasis model of insulin resistance.

**Table 4** shows multiple linear regression analysis between plasma asprosin levels and clinical parameters. Multiple linear regression analysis indicated that plasma asprosin levels were independently associated with HOMA-IR and TG/HDL cholesterol ratio (Adjusted R^2^ = 0.37, *p* < 0.001). Mediating effects analysis was conducted to examine whether the association between BMI SDS and HOMA-IR was mediated by asprosin (**Figure 4**). The size of the total effect of BMI SDS on HOMA-IR was estimated as β = 0.40 (where β is the standardized coefficient). The direct effect of BMI SDS on HOMA-IR was estimated as β = 0.27, accounting for 68% of the total effect. The indirect effect of BMI SDS on HOMA-IR was estimated as β = 0.13, accounting for 32% of the total effect. The results of the mediation analysis indicated that asprosin appeared to play a mediating role in the relationship between obesity and insulin resistance.

**Table 4:** Simple and multiple linear regression analyses of variables associated with plasma asprosin levels.

	Simple linear regression				Multiple linear regression^a^			
	Unstandardized coefficients		Standardized coefficients		Unstandardized coefficients		Standardized coefficients	
Variables	B	SE	β	*p*-value	B	SE	β	*p*-value
Age	-2.41	1.39	-0.19	0.09	-1.43	1.26	-0.11	0.26
Male sex	7.19	5.58	0.14	0.20	9.72	5.05	0.19	0.06
BMI SDS	13.52	4.12	0.34	0.002	0.64	4.26	0.02	0.88
HOMA-IR	3.21	0.68	0.47	<0.001	2.87	0.68	0.42	<0.001
TG/HDL cholesterol ratio	8.95	2.17	0.42	<0.001	8.15	1.95	0.38	<0.001

a Adjusted R^2^ = 0.37, *p* < 0.001.

BMI SDS, body mass index standard deviation score; HDL, high-density lipoprotein; HOMA-IR, homeostasis model assessment-insulin resistance; SE, standard error; TG, triglycerides.

## 4 Discussion

We evaluated the associations between asprosin levels and anthropometric and metabolic parameters in 109 Korean children and adolescents. To our knowledge, this study is one of few to assess the associations of asprosin with obesity and insulin resistance in children, and it is the first in Korean children. We demonstrated significantly higher plasma asprosin levels in the obese group than in the controls and in the insulin resistance group than in the non-insulin resistance group. Plasma asprosin levels were positively correlated with BMI SDS, glucose, insulin, HOMA-IR, and TG and negatively correlated with HDL cholesterol.

Our research has uncovered a significant positive correlation between asprosin levels and BMI in children and adolescents. These findings are consistent with two pediatric studies (17, 19) but not with two others (18, 20). This discrepancy may be partly explained by differences in the study population and the experimental methods for quantification of asprosin. For example, two studies of Chinese children showed contrasting results regarding the correlation between BMI and asprosin levels. One study showed a positive correlation (17), while the other showed a negative one (18). BMI and HOMA-IR in obese children were higher in the former study (17) than in the latter study (18). It should be noted that different ELISA kits were used in all five studies, including our study and the four previous studies (17–20). In the present study, plasma asprosin levels were higher in children and adolescents with IR than in those without IR. Consistent with our findings, previous adult studies showed that asprosin levels were higher in the insulin resistance group than in the non-insulin resistance group (3, 5–7). Considering the association of asprosin with obesity and insulin resistance, asprosin may be a novel target molecule in preventing the development of child obesity and metabolic diseases.

The relationships between circulating asprosin levels and lipid parameters have been well described in adult studies (9, 10, 26). Li et al. found that asprosin levels in female adults positively correlated with total cholesterol, LDL cholesterol, and TG and negatively correlated with HDL cholesterol (26). Two other studies in adults with type 2 DM reported a positive correlation between asprosin levels and TG (9, 10). However, these correlations have yet to be evident in children (17–20). Notably, in this study, plasma asprosin levels were independently associated with the TG/HDL cholesterol ratio, which reflects the effect of insulin resistance on lipid metabolism (27). The TG/HDL cholesterol ratio has been recognized as a marker of structural vascular changes and insulin resistance in obese children (27–29). Our data suggest that plasma asprosin levels may play a role in insulin resistance and lipid metabolism in obese children. Further studies should be aimed at evaluating these issues.

In the current study, no correlation was found between plasma asprosin levels and Tanner stages in boys and girls, consistent with the findings of Long et al. (18). Several previous studies have shown conflicting results regarding the association between sex and circulating asprosin levels. We demonstrated no difference in plasma asprosin levels between boys and girls, consistent with two previous studies (17, 19). Some studies have shown lower asprosin levels in males than in females (7, 18, 20). Long et al. documented asprosin as being lower in boys than in girls in the obese group but not in the control group (18). However, another study demonstrated lower serum asprosin levels in boys than in girls (20).

This study has some limitations. First, our findings are based on a single-center cohort, and all subjects were referred to a pediatric endocrinologist. Therefore, the results might not be fully representative of the general population. Second, body fat mass, muscle mass, diet, and exercise status were unknown, and the effect of these factors on asprosin level remains to be determined. Third, HOMA-IR was used to determine IR, but clamp methods are considered the gold standard for evaluating IR. Lastly, the ELISA kit used has, to the best of our knowledge, not been evaluated by independent researchers, including ourselves.

In conclusion, this study identified significant correlations between elevated levels of asprosin and obesity and insulin resistance in Korean children and adolescents. Plasma asprosin levels were associated with parameters of glucose and lipid metabolism. Our findings suggest a potential role for asprosin in obesity and metabolic disorders, indicating the need for further research to better understand its function.

## Data Availability

The original contributions presented in the study are included in the article/supplementary material. Further inquiries can be directed to the corresponding author.
